# A CNN‐based denoising method trained with images acquired with electron density phantoms for thin‐sliced coronary artery calcium scans

**DOI:** 10.1002/acm2.14287

**Published:** 2024-02-12

**Authors:** Ching‐Ching Yang, Kuei‐Yuan Hou

**Affiliations:** ^1^ Department of Medical Imaging and Radiological Sciences Kaohsiung Medical University Kaohsiung Taiwan; ^2^ Department of Medical Research Kaohsiung Medical University Hospital Kaohsiung Taiwan; ^3^ Department of Radiology Cathay General Hospital Taipei Taiwan

**Keywords:** CNN‐based denoising method, coronary artery calcium scan, partial volume effect

## Abstract

**Purpose:**

This work proposed a convolutional neural network (CNN)‐based method trained with images acquired with electron density phantoms to reduce quantum noise for coronary artery calcium (CAC) scans reconstructed with slice thickness less than 3 mm.

**Methods:**

A DenseNet model was used to estimate quantum noise for CAC scans reconstructed with slice thickness of 0.5, 1.0 and 1.5 mm. Training data was acquired using electron density phantoms in three different sizes. The label images of the CNN model were real noise maps, while the input images of the CNN model were pseudo noise maps. Image denoising was conducted by subtracting the CNN output images from thin‐sliced CAC scans. The efficacy of the proposed method was verified through both phantom study and patient study.

**Results:**

By means of phantom study, the proposed method was proven effective in reducing quantum noise in CAC scans reconstructed with 1.5‐mm slice thickness without causing significant texture change or variation in HU values. With regard to patient study, calcifications were more clear on the denoised CAC scans reconstructed with slice thickness of 0.5, 1.0 and 1.5 mm than on 3‐mm slice images, while over‐smooth changes were not observed in the denoised CAC scans reconstructed with 1.5‐mm slice thickness.

**Conclusion:**

Our results demonstrated that the electron density phantoms can be used to generate training data for the proposed CNN‐based denoising method to reduce quantum noise for CAC scans reconstructed with 1.5‐mm slice thickness. Because anthropomorphic phantom is not a necessity, our method could make image denoising more practical in routine clinical practice.

## 1 INTRODUCTION

Partial volume averaging is critical for object that are at the voxel resolution or less, so it is particularly problematic in CT scans for the diagnosis of coronary artery disease, such as coronary CT angiography (CCTA) and coronary artery calcium (CAC) scans.^1^, ^2^, ^3^ In CCTA, images are usually reconstructed with a slice thickness of 0.5−0.6 mm, while the slice thickness used in routine CAC scoring is 3 mm.^4^, ^5^, ^6^ The main coronary arteries are usually between 3 to 4 mm in diameter, so the visibility of coronary artery calcifications can be improved by using a thinner slice thickness.^7^, ^8^, ^9^, ^10^, ^11^, ^12^ However, quantum noise increases with reduced slice thickness. For conventional FBP images, the standard deviation (SD) in Hounsfield units (HU) due to Poisson noise is proportional to $\sqrt{1/(\mathrm{slice}\;\mathrm{thickness}\times \mathrm{mAs})}$.^13^ A higher mAs can be used to maintain the same image quality for thin slice reconstruction, but it would result in a higher radiation dose. Because it is imperative to keep radiation dose as low as reasonably achievable (ALARA), CT image denoising is an alternative to reduce the quantum noise due to thin slice reconstruction. Generally, the process of noise suppression is known as image denoising, which has been well studied over the past several decades.^14^, ^15^ Recently, convolutional neural network (CNN)‐based denoising methods, which attempt to learn a mapping function by optimizing a loss function on a training set that contains degraded‐clean image pairs, have been developed rapidly.^16^, ^17^, ^18^, ^19^ Hou et al. have reported that Densely connected convolutional network (DenseNet) can be used to reduce quantum noise in CAC scans reconstructed with 1.5‐mm slice thickness, where the model training was conducted by using datasets acquired with anthropomorphic thorax phantoms instead of actual patient scans to avoid any bias caused by partial volume averaging.^20^ The anthropomorphic thorax phantoms could mimic the CT density and attenuation properties of human thorax with variable calcium inserts, but they are not widely available compared with the electron density phantom. In order to make image denoising more practical in routine clinical practice, this work proposed a CNN‐based method trained with images acquired with electron density phantoms to reduce quantum noise for CAC scans reconstructed with slice thickness less than 3 mm.

## 2 METHODS

### 2.1 CAC scan

All images were obtained from a 320‐detector row CT system (Aquilion One, Canon, Otawara, Japan). In our department, routine CAC scans are performed using tube current modulation at 120 kVp, where the image quality reference parameter was set as 55 to adjust tube current with a range of 40−300 mA. The gantry rotation speed was 0.35 s/rotation. Images were reconstructed using FBP with soft tissue kernel (FC12). All reconstructions employed a matrix size of 512 × 512 and 22‐cm display field‐of‐view. Phantom studies were carried out using gated protocols, wherein a cardiac simulator was employed to simulate an ECG signal with a mean heart rate of 60 bpm.

### 2.2 Electron density phantoms

**FIGURE 1:**
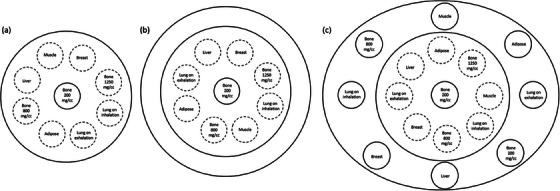
Electron density phantoms used for model training: (a) CIRS_S, (b) CIRS_M, (c) CIRS_L.

The electron density phantoms (Model 062 M; CIRS, Norfolk, VA, USA), including CIRS_S (180 mm in diameter), CIRS_M (230 mm in diameter) and CIRS_L (320 mm × 270 mm), were used to generate data for model training (Figure 1). CIRS_S houses 9 rod inserts simulating lung, adipose, breast, muscle, liver, and bone with calcium hydroxyapatite (HA) density of 200, 800, and 1250 mg/cc. The rods were 30 mm in diameter and 50 mm in length. CIRS_M was constructed by covering CIRS_S with a 2.5‐cm‐thick bolus (Superflab Bolus; Radiation Products Design Inc, Albertville, MN, USA). CIRS_L consists of CIRS_S and an additional annulus made of soft tissue equivalent epoxy resin. CAC scan was first performed with a phantom housing all the rod inserts. Next, CAC scan was performed after removing two rods from the phantom. This process was repeated until all the rods marked with dashed circles in Figure 1 were all removed, so a total of 15 data sets were obtained for CNN training (3 phantoms × 5 scans). The volume CT dose index (CTDI_vol_) was 1.1, 2.0, 9.7 mGy for CIRS_S, CIRS_M and CIRS_L, respectively, which were resulted from a CAC scan with tube current of 40, 80, 300 mA, respectively.

### 2.3 CNN model

**FIGURE 2:**
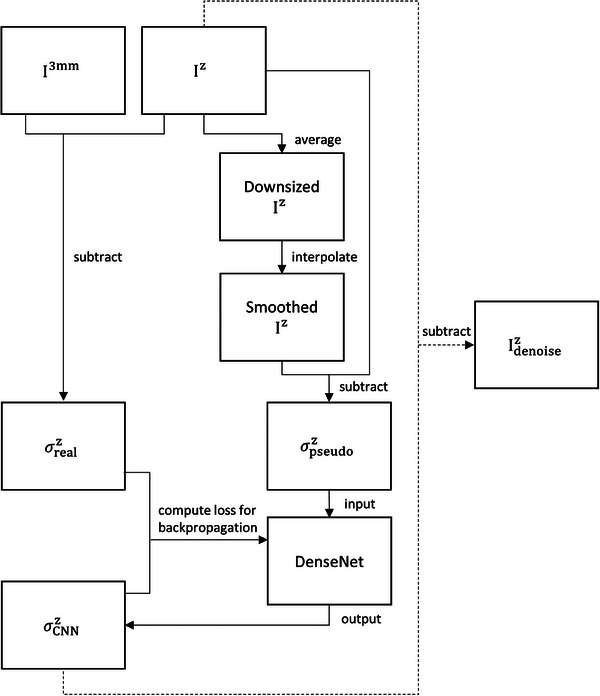
Flowchart of the proposed CNN‐based denoising method (*z* = 0.5, 1.0 or 1.5 mm).

The DenseNet model proposed by Tong et al. was used in this work to estimate quantum noise for CAC scans reconstructed with slice thickness of 0.5 mm (I^0.5mm^), 1.0 mm (I^1.0mm^) and 1.5 mm (I^1.5mm^).^21^ The label image of the CNN model was the difference between 3‐mm sliced CAC scan (I^3mm^)) and I^z^ (*z* = 0.5, 1.0 or 1.5 mm), that is, the real noise map ($\sigma_{\mathrm{real}}^{z}$). The input image of the CNN model was a pseudo noise map ($\sigma_{\mathrm{pseudo}}^{z}$), which was the difference between the original I^z^ and a smoothed I^z^. To create the smoothed I^z^, I^z^ was averaged every 2 × 2 pixels to reduce the matrix size from 512 × 512 to 256 × 256 and then interpolated by bi‐cubic interpolation to increase the matrix size from 256 × 256 to 512 × 512. The root mean square error (RMSE) was the loss function adopted to minimize the difference between CNN generated noise map ($\sigma_{\mathrm{CNN}}^{z}$) and $\sigma_{\mathrm{real}}^{z}$. By utilizing RMSE as the loss function, the preference is given to achieving a high peak signal‐to‐noise ratio (PSNR). The filter weights for each layer were initialized using the MSRA (Microsoft Research Asia) filler technique, while all biases were initialized to zero. The models were trained using the Adam (adaptive moment estimation) optimizer with a mini‐batch size of 32, a learning rate of 0.0001, a momentum of 0.9, and a weight decay of 0.0001. The training datasets consisted of approximately 70 020 sub‐images, each measuring 25 × 25 pixels with a stride of 25 pixels. These sub‐images were shuffled by applying random permutation, serving as both input and label images. The CNN models were built by using Caffe (Convolutional Architecture for Fast Feature Embedding) CNN platform on an Ubuntu server. Figure 2 shows the workflow of the proposed CNN‐based denoising method, where $I_{\mathrm{denoise}}^{z}$ was the denoising results for I^z^, which was obtained by subtracting $\sigma_{\mathrm{CNN}}^{z}$ from I^z^.

### 2.4 Anthropomorphic thorax phantoms

**FIGURE 3:**
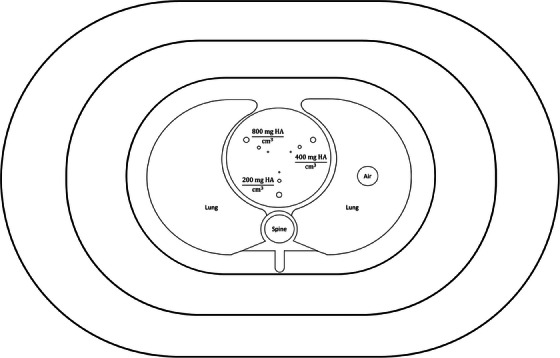
Anthropomorphic thorax phantoms used for model validation.

The anthropomorphic thorax phantoms (Thorax‐CCI, QRM GmbH, Möhrendorf, Germany), including QRM_S (300 mm × 200 mm), QRM_M (350 mm × 250 mm) and QRM_L (400 mm × 300 mm), were used to verify the efficacy of the proposed image denoising method (Figure 3). QRM_S consists of an anthropomorphic phantom body and a calibration insert, where the calibration insert contains nine cylindrical calcifications that are in three different sizes (1, 3, 5 mm in diameter and height) and three different HA densities (200, 400, and 800 mg/cm^3^). QRM_M consists of QRM_S and one extension ring, while QRM_L consists of QRM_S and two extension rings. The CTDI_vol_ was 1.1, 1.4, 4.2 mGy for QRM_S, QRM_M and QRM_L, respectively, which were resulted from a CAC scan with tube current of 40, 50, 140 mA.

### 2.5 Data analysis for phantom validation

RMSE and PSNR were calculated to quantify the difference between $I^{3\;\mathrm{mm}}$ and $I_{\mathrm{denoise}}^{z}$ (*z* = 0.5, 1.0 and 1.5 mm) over the whole image, defined as

(1) $$\mathrm{RMSE}\;=\sqrt{\frac{\sum_{i=1}^{k}{\left(I^{3\mathrm{mm}}\;-{\;I}_{\mathrm{denoise}}^{z}\right)}^{2}}{k}}\;$$

where k is the number of voxels within the whole image;

(2) $$\mathrm{PSNR}\;=\;20\cdot \log\left(\frac{HU_{\max}}{\mathrm{RMSE}}\right)$$

where HU_max_ is the maximum HU value of the image. To investigate the quality and reliability of CT images within local sub‐regions, region of interest (ROI) analysis was conducted to calculate the mean and SD within ROIs. Two‐sample *t*‐test was used to compare HU values within ROIs measured from $I^{3\;\mathrm{mm}}$ with those from I^z^ and $I_{\mathrm{denoise}}^{z}$. A *p* < 0.01 was used to determine statistical significance. In the case of electron density phantoms, a circular ROI was positioned at the center of the rod insert. Regarding the thorax phantoms, a threshold of 130 HU was employed to identify ROIs associated with calcification. In addition to the calcification ROIs, a background ROI was delineated on the calibration insert. Dice similarity coefficient was used to compare the calcification pixels in $I^{3\;\mathrm{mm}}$ with those in I^z^ and $I_{\mathrm{denoise}}^{z}$. Due to the inability to detect 1‐mm‐diameter calcifications in 3‐mm slice images, these calcifications were excluded from data analysis.

### 2.6 Patient study

A retrospective analysis of patients who underwent CAC scans at our institution was conducted to evaluate the efficacy of the proposed method on real patients. Permission to access patient data was obtained following approval from the Institutional Human Research Ethics Committee. The Agatston score was calculated by multiplying the calcification area with a specific weighting factor, which is 1 for calcifications ranging from 130−199 HU, 2 for 200−299 HU, 3 for 300−399 HU and 4 for calcifications exceeding 400 HU. Therefore, the number of calcification pixels assigned for different weighting factors was measured on I^3mm^ and $I_{\mathrm{denoise}}^{z}$ (*z* = 0.5, 1.0, 1.5 mm) of clinical patients.

## 3 RESULTS

### 3.1 Validation with electron density phantoms

**FIGURE 4:**
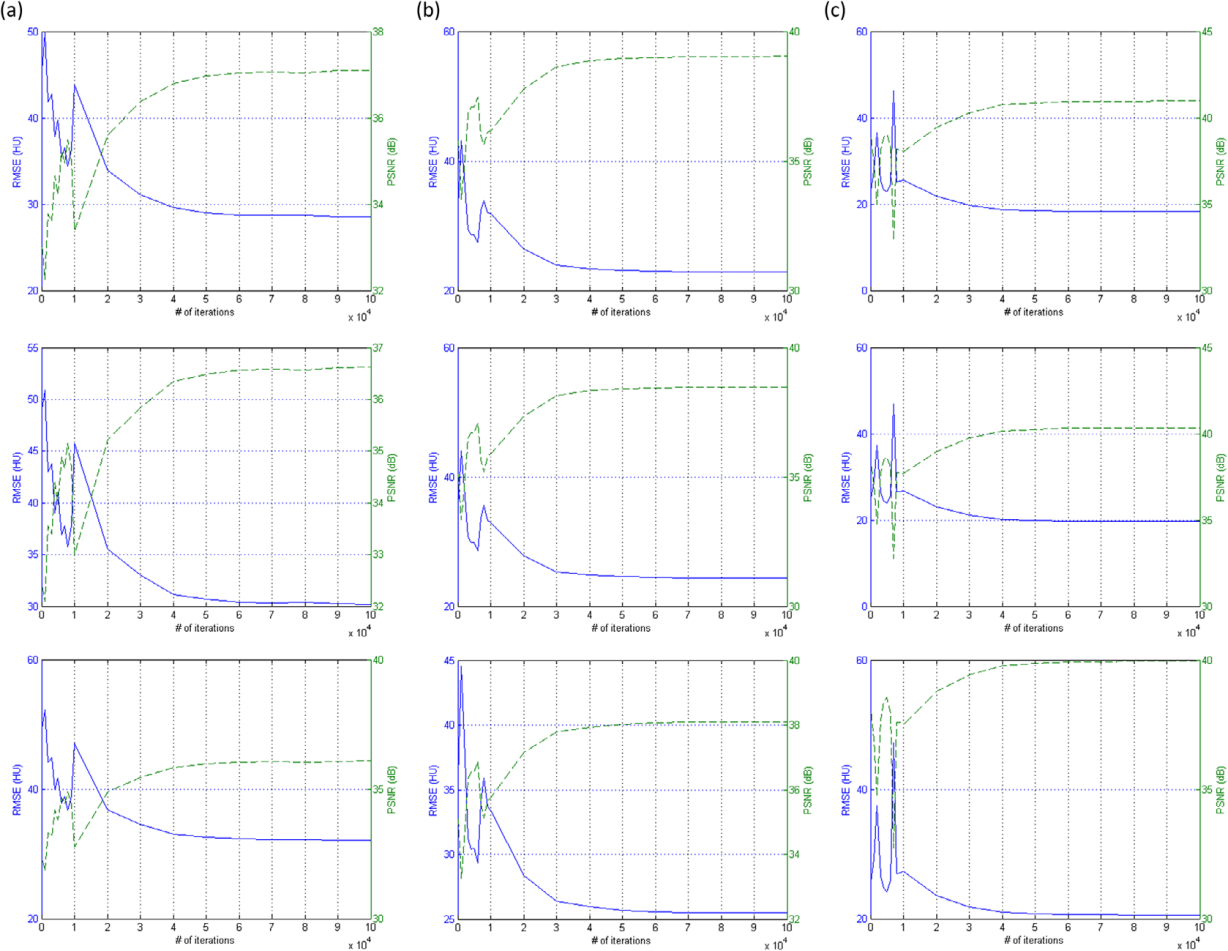
RMSE (solid line, left axis) and PSNR (dashed line, right axis) between (a) $I^{3\;\mathrm{mm}}$ and $I_{\mathrm{denoise}}^{0.5\;\mathrm{mm}}$, (b) $I^{3\;\mathrm{mm}}$ and $I_{\mathrm{denoise}}^{1.0\;\mathrm{mm}}$, (c) $I^{3\;\mathrm{mm}}$ and $I_{\mathrm{denoise}}^{1.5\;\mathrm{mm}}$ under different training iterations for CIRS_S, CIRS_M, CIRS_L (from top to bottom).

**FIGURE 5:**
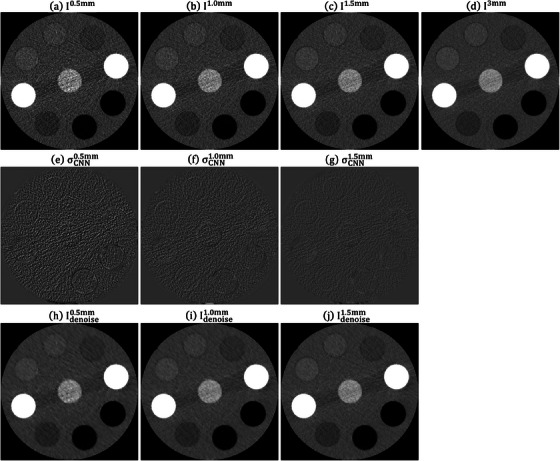
Axial plane of (a) $I^{0.5\;\mathrm{mm}}$, (b) $I^{1.0\;\mathrm{mm}}$, (c) $I^{1.5\;\mathrm{mm}}$, (d) $I^{3\;\mathrm{mm}}$, (e) $\sigma_{\mathrm{CNN}}^{0.5\;\mathrm{mm}}$, (f) $\sigma_{\mathrm{CNN}}^{1.0\;\mathrm{mm}}$, (g) $\sigma_{\mathrm{CNN}}^{1.5\;\mathrm{mm}}$, (h) $I_{\mathrm{denoise}}^{0.5\;\mathrm{mm}}$, (i) $I_{\mathrm{denoise}}^{1.0\;\mathrm{mm}}$, (j) $I_{\mathrm{denoise}}^{1.5\;\mathrm{mm}}$ for CIRS_S (window level/window width = 0/1000 HU for (a)−(d), (h)−(j) and 0/340 HU for (e)−(g)). HU, Hounsfield units.

**FIGURE 6:**
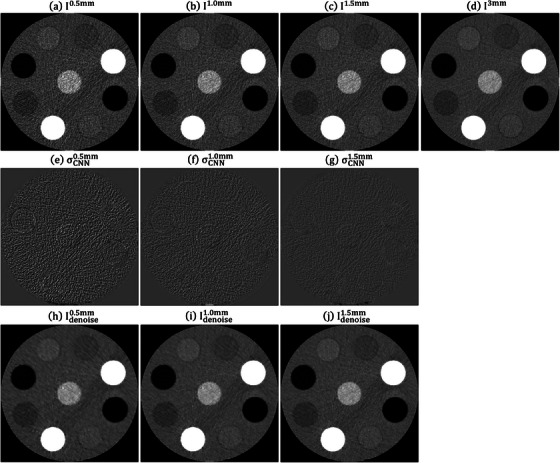
Axial plane of (a) $I^{0.5\;\mathrm{mm}}$, (b) $I^{1.0\;\mathrm{mm}}$, (c) $I^{1.5\;\mathrm{mm}}$, (d) $I^{3\;\mathrm{mm}}$, (e) $\sigma_{\mathrm{CNN}}^{0.5\;\mathrm{mm}}$, (f) $\sigma_{\mathrm{CNN}}^{1.0\;\mathrm{mm}}$, (g) $\sigma_{\mathrm{CNN}}^{1.5\;\mathrm{mm}}$, (h) $I_{\mathrm{denoise}}^{0.5\;\mathrm{mm}}$, (i) $I_{\mathrm{denoise}}^{1.0\;\mathrm{mm}}$, (j) $I_{\mathrm{denoise}}^{1.5\;\mathrm{mm}}$ for CIRS_M (window level/window width = 0/1000 HU for (a)−(d), (h)−(j) and 0/340 HU for (e)−(g)). HU, Hounsfield units.

**FIGURE 7:**
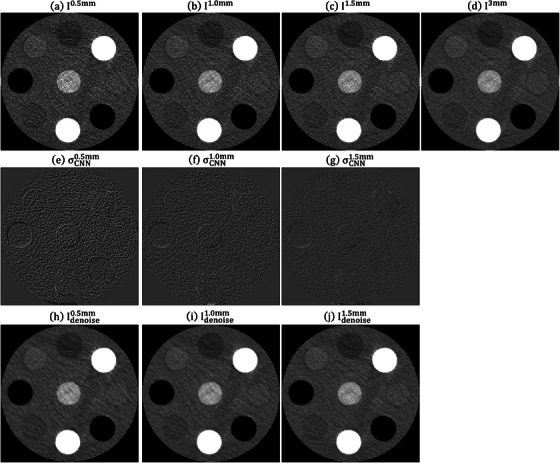
Axial plane of (a) $I^{0.5\;\mathrm{mm}}$, (b) $I^{1.0\;\mathrm{mm}}$, (c) $I^{1.5\;\mathrm{mm}}$, (d) $I^{3\;\mathrm{mm}}$, (e) $\sigma_{\mathrm{CNN}}^{0.5\;\mathrm{mm}}$, (f) $\sigma_{\mathrm{CNN}}^{1.0\;\mathrm{mm}}$, (g) $\sigma_{\mathrm{CNN}}^{1.5\;\mathrm{mm}}$, (h) $I_{\mathrm{denoise}}^{0.5\;\mathrm{mm}}$, (i) $I_{\mathrm{denoise}}^{1.0\;\mathrm{mm}}$, (j) $I_{\mathrm{denoise}}^{1.5\;\mathrm{mm}}$ for CIRS_L (window level/window width = 0/1000 HU for (a)−(d), (h)−(j) and 0/340 HU for (e)−(g)). HU, Hounsfield units.

Figure 4 shows the RMSE and PSNR between $I^{3\;\mathrm{mm}}$ and $I_{\mathrm{denoise}}^{z}$ (*z* = 0.5, 1.0 and 1.5 mm) under different training iterations for CIRS_S, CIRS_M and CIRS_L. After conducting 80 000 iterations, no significant enhancement in RMSE and PSNR was observed. Therefore, for this study, the CNN model trained for 10 0000 iterations was utilized. Figure 5 demonstrates an axial plane of $I^{z}$, $\sigma_{\mathrm{CNN}}^{z}$, $I_{\mathrm{denoise}}^{z}$ (*z* = 0.5, 1.0, 1.5 mm) and $I^{3\;\mathrm{mm}}$ for CIRS_S. Figure 6 presents the corresponding outcomes for CIRS_M, while Figure 7 displays the results for CIRS_L. Table 1 summaries the analysis results of HU values for rod inserts in three electron density phantoms simulating various tissue materials on $I^{3\;\mathrm{mm}}$, $I^{z}$ and $I_{\mathrm{denoise}}^{z}$ (*z* = 0.5, 1.0, 1.5 mm). The *p*‐values were acquired by conducting a two‐sample *t*‐test to assess the disparity in HU values between $I^{3\;\mathrm{mm}}$ and the remaining images.

**TABLE 1:** Analysis results of HU values for rod inserts in CIRS_S, CIRS_M, CIRS_L.

		$\;I^{3\;\mathrm{mm}}$		$\;I^{0.5\;\mathrm{mm}}$			$\;I^{1.0\;\mathrm{mm}}$			$\;I^{1.5\;\mathrm{mm}}$			${\;I}_{\mathrm{denoise}}^{0.5\;\mathrm{mm}}$			${\;I}_{\mathrm{denoise}}^{1.0\;\mathrm{mm}}$			${\;I}_{\mathrm{denoise}}^{1.5\;\mathrm{mm}}$		
		Mean (HU)	SD (HU)	Mean (HU)	SD (HU)	*p*‐ Value	Mean (HU)	SD (HU)	*p‐*Value	Mean (HU)	SD (HU)	*p*‐ Value	Mean (HU)	SD (HU)	*p*‐ Value	Mean (HU)	SD (HU)	*p* ‐Value	Mean (HU)	SD (HU)	*p*‐ Value
CIRS_S	Lung inhale	−829.59	22.87	−828.43	42.77	0.20	−829.10	37.27	0.55	−829.92	30.86	0.65	−828.58	19.06	0.07	−829.39	20.35	0.73	−830.24	21.01	0.26
	Lung exhale	−481.16	27.53	−479.23	52.64	0.09	−478.90	41.58	0.02	−480.08	34.64	0.20	−479.72	22.45	0.03	−480.01	23.30	0.09	−480.93	23.23	0.74
	Adipose	−72.86	27.69	−72.38	57.74	0.69	−74.23	45.30	0.17	−73.19	37.54	0.71	−71.60	25.55	0.08	−74.07	24.45	0.08	−73.41	25.16	0.44
	Breast	−25.93	27.80	−25.80	52.30	0.91	−24.65	42.30	0.18	−25.45	35.28	0.57	−25.75	27.05	0.80	−25.68	24.33	0.72	−25.97	24.80	0.95
	Muscle	56.77	25.29	57.29	51.97	0.63	56.33	41.15	0.63	56.23	34.08	0.50	58.10	27.19	0.06	56.39	23.61	0.56	56.20	23.60	0.38
	Liver	71.83	24.31	71.71	50.61	0.91	72.93	42.26	0.23	72.22	34.00	0.62	72.91	22.06	0.08	72.75	23.95	0.15	72.08	23.24	0.69
	Bone 200 mg/cc	257.64	37.71	256.16	86.57	0.41	257.39	63.40	0.86	258.22	51.67	0.63	256.82	45.07	0.46	256.98	36.97	0.51	257.22	36.13	0.67
	Bone 800 mg/cc	1037.05	40.51	1035.98	69.16	0.48	1036.66	60.24	0.77	1036.88	50.74	0.89	1036.06	34.70	0.32	1036.62	33.58	0.66	1036.24	34.80	0.42
	Bone 1250 mg/cc	1577.16	42.04	1575.85	74.82	0.42	1576.10	63.77	0.46	1576.34	55.37	0.53	1577.75	38.77	0.58	1576.87	40.47	0.79	1576.14	41.08	0.36
CIRS_M	Lung inhale	−822.11	25.07	−822.83	45.57	0.46	−822.48	38.68	0.67	−822.03	34.02	0.92	−822.17	20.22	0.92	−822.95	21.21	0.17	−822.39	22.92	0.66
	Lung exhale	−467.80	27.58	−465.65	56.58	0.07	−467.15	43.44	0.50	−467.10	36.02	0.41	−467.44	25.06	0.60	−468.91	23.07	0.10	−468.13	23.95	0.63
	Adipose	−68.26	32.70	−69.11	77.37	0.59	−69.31	53.95	0.38	−68.22	45.87	0.97	−69.14	32.24	0.31	−69.50	28.29	0.13	−69.40	30.10	0.17
	Breast	−22.65	29.55	−23.08	54.24	0.71	−23.87	43.82	0.22	−23.74	38.33	0.23	−22.56	28.80	0.91	−24.18	25.63	0.04	−24.08	26.73	0.06
	Muscle	44.62	31.71	43.85	64.57	0.57	44.71	52.47	0.94	44.92	44.07	0.77	44.80	27.77	0.82	44.48	28.53	0.86	44.24	29.46	0.64
	Liver	73.83	28.49	72.73	53.99	0.34	73.37	46.03	0.65	73.00	39.17	0.37	73.70	23.38	0.86	73.10	24.42	0.30	72.92	25.66	0.21
	Bone 200 mg/cc	274.56	40.76	270.77	78.54	0.02	272.09	60.93	0.07	273.71	52.89	0.07	271.47	35.36	*	271.85	33.58	0.01	273.79	36.04	0.05
	Bone 800 mg/cc	955.10	40.85	957.29	89.16	0.23	955.01	66.13	0.95	955.68	56.79	0.66	957.78	43.44	0.02	955.45	37.76	0.74	954.91	39.23	0.86
	Bone 1250 mg/cc	1532.88	43.65	1531.80	74.32	0.50	1534.10	65.51	0.41	1532.20	57.97	0.62	1531.98	38.19	0.41	1534.40	39.55	0.17	1532.66	42.50	0.84
CIRS_L	Lung inhale	−792.05	29.97	−793.54	55.86	0.21	−792.75	44.81	0.49	−792.91	39.34	0.36	−792.95	28.41	0.25	−793.41	26.14	0.07	−793.37	27.51	0.09
	Lung exhale	−463.03	27.22	−465.21	51.81	0.05	−462.96	41.99	0.94	−463.78	36.39	0.38	−464.85	26.12	0.01	−464.04	24.09	0.14	−464.51	25.71	0.04
	Adipose	−64.57	38.76	−65.97	68.69	0.34	−64.51	57.03	0.97	−64.77	49.76	0.86	−65.56	39.68	0.34	−64.81	38.04	0.81	−65.31	37.92	0.47
	Breast	−1.10	30.99	−1.87	60.90	0.55	−2.85	49.94	0.11	−2.02	41.96	0.35	−1.29	33.41	0.82	−3.25	30.31	0.01	−2.43	30.31	0.10
	Muscle	54.83	38.07	54.39	68.21	0.76	55.93	58.08	0.40	54.40	50.90	0.72	55.77	33.43	0.33	55.54	33.89	0.46	53.51	35.79	0.18
	Liver	78.95	34.16	79.10	57.33	0.91	79.79	51.67	0.47	79.25	43.50	0.77	79.93	28.65	0.25	78.84	30.72	0.89	78.35	31.28	0.49
	Bone 200 mg/cc	289.87	44.79	290.68	87.91	0.66	288.80	65.10	0.47	288.44	57.64	0.30	291.68	47.79	0.14	288.43	41.48	0.21	287.64	42.96	0.06
	Bone 800 mg/cc	924.29	42.09	922.85	73.96	0.37	924.56	57.23	0.84	923.94	50.75	0.78	922.84	38.76	0.18	923.68	36.31	0.56	922.71	37.81	0.14
	Bone 1250 mg/cc	1437.49	48.52	1437.21	85.16	0.88	1437.29	70.14	0.90	1438.30	62.09	0.58	1436.52	42.36	0.42	1435.63	41.62	0.12	1436.71	45.38	0.53

Abbreviations: HU, Hounsfield units; SD, standard deviation.

### 3.2 Validation with anthropomorphic thorax phantoms

**FIGURE 8:**
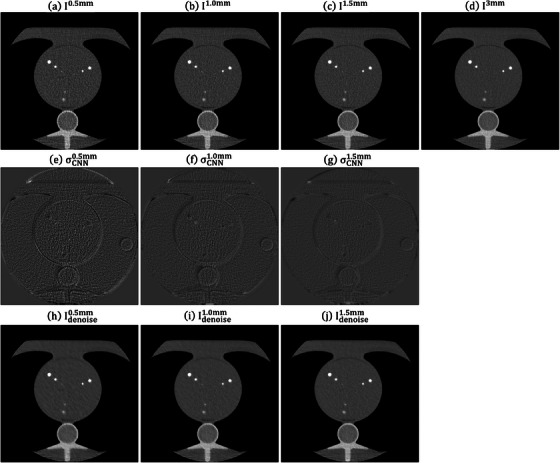
Axial plane of (a) $I^{0.5\;\mathrm{mm}}$, (b) $I^{1.0\;\mathrm{mm}}$, (c) $I^{1.5\;\mathrm{mm}}$, (d) $I^{3\;\mathrm{mm}}$, (e) $\sigma_{\mathrm{CNN}}^{0.5\;\mathrm{mm}}$, (f) $\sigma_{\mathrm{CNN}}^{1.0\;\mathrm{mm}}$, (g) $\sigma_{\mathrm{CNN}}^{1.5\;\mathrm{mm}}$, (h) $I_{\mathrm{denoise}}^{0.5\;\mathrm{mm}}$, (i) $I_{\mathrm{denoise}}^{1.0\;\mathrm{mm}}$, (j) $I_{\mathrm{denoise}}^{1.5\;\mathrm{mm}}$ for QRM_S (window level/window width = 0/1000 HU for (a)−(d), (h)−(j) and 0/340 HU for (e)−(g)). HU, Hounsfield units.

**FIGURE 9:**
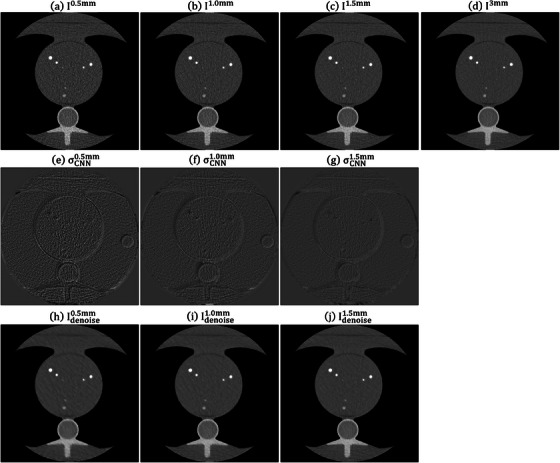
Axial plane of (a) $I^{0.5\;\mathrm{mm}}$, (b) $I^{1.0\;\mathrm{mm}}$, (c) $I^{1.5\;\mathrm{mm}}$, (d) $I^{3\;\mathrm{mm}}$, (e) $\sigma_{\mathrm{CNN}}^{0.5\;\mathrm{mm}}$, (f) $\sigma_{\mathrm{CNN}}^{1.0\;\mathrm{mm}}$, (g) $\sigma_{\mathrm{CNN}}^{1.5\;\mathrm{mm}}$, (h) $I_{\mathrm{denoise}}^{0.5\;\mathrm{mm}}$, (i) $I_{\mathrm{denoise}}^{1.0\;\mathrm{mm}}$, (j) $I_{\mathrm{denoise}}^{1.5\;\mathrm{mm}}$ for QRM_M (window level/window width = 0/1000 HU for (a)−(d), (h)−(j) and 0/340 HU for (e)−(g)). HU, Hounsfield units.

**FIGURE 10:**
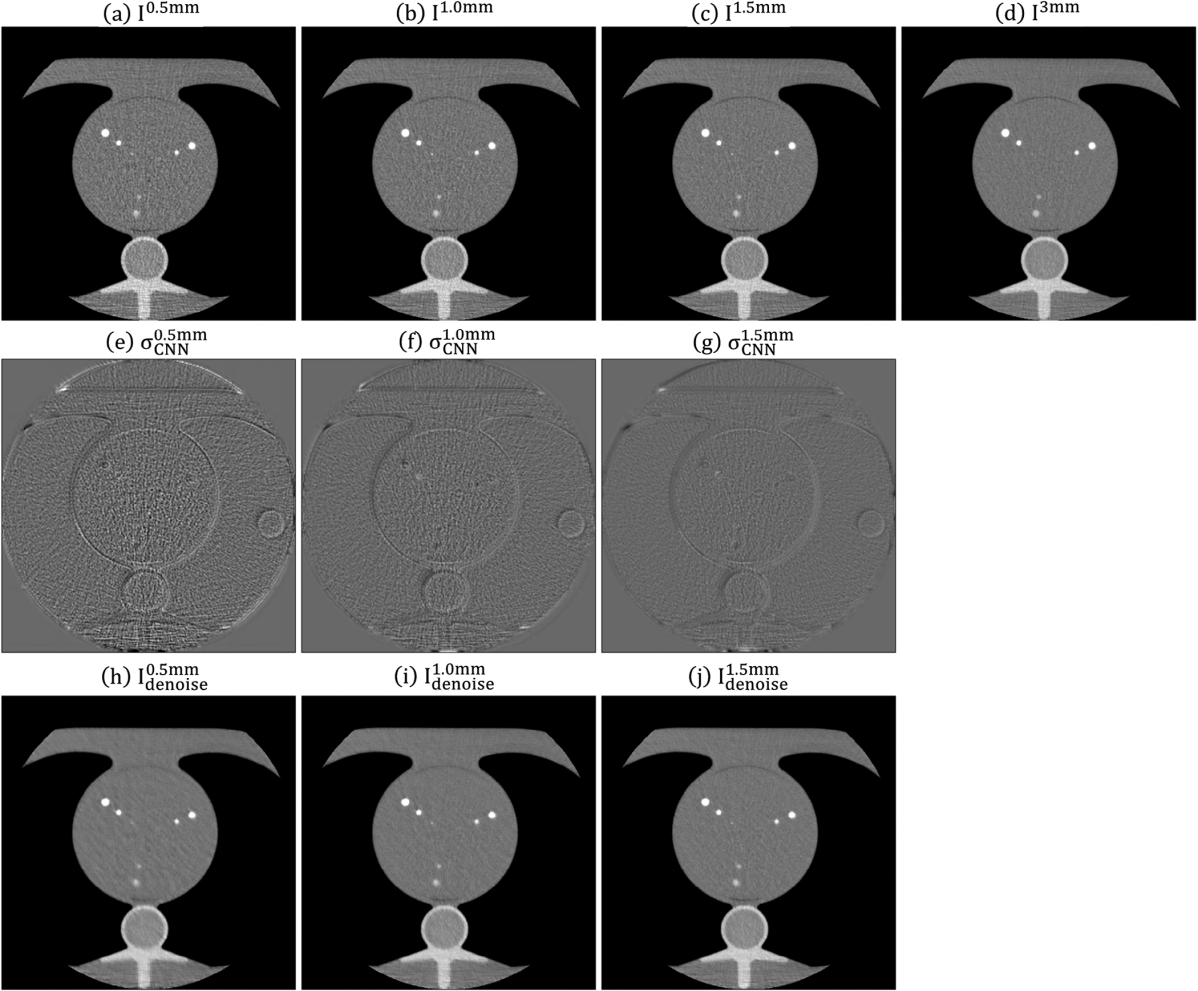
Axial plane of (a) $I^{0.5\;\mathrm{mm}}$, (b) $I^{1.0\;\mathrm{mm}}$, (c) $I^{1.5\;\mathrm{mm}}$, (d) $I^{3\;\mathrm{mm}}$, (e) $\sigma_{\mathrm{CNN}}^{0.5\;\mathrm{mm}}$, (f) $\sigma_{\mathrm{CNN}}^{1.0\;\mathrm{mm}}$, (g) $\sigma_{\mathrm{CNN}}^{1.5\;\mathrm{mm}}$, (h) $I_{\mathrm{denoise}}^{0.5\;\mathrm{mm}}$, (i) $I_{\mathrm{denoise}}^{1.0\;\mathrm{mm}}$, (j) $I_{\mathrm{denoise}}^{1.5\;\mathrm{mm}}$ for QRM_L (window level/window width = 0/1000 HU for (a)−(d), (h)−(j) and 0/340 HU for (e)−(g)). HU, Hounsfield units.

**FIGURE 11:**
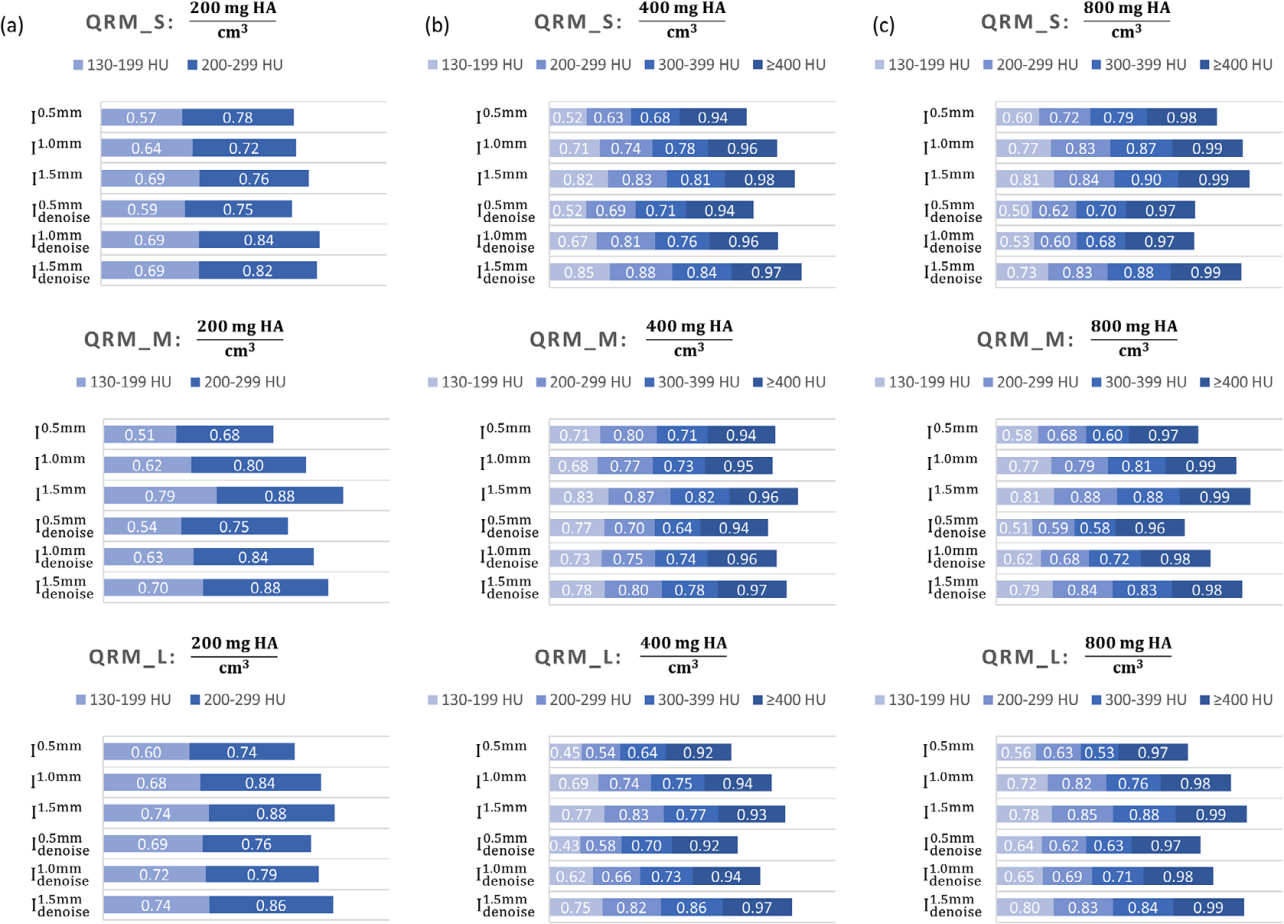
Dice similarity coefficients calculated by comparing calcification pixels in $I^{3\;\mathrm{mm}}$ with those in $I^{0.5\;\mathrm{mm}}$, $I^{1.0\;\mathrm{mm}}$, $I^{1.5\;\mathrm{mm}}$, $I_{\mathrm{denoise}}^{0.5\;\mathrm{mm}}$, $I_{\mathrm{denoise}}^{1.0\;\mathrm{mm}}$, $I_{\mathrm{denoise}}^{1.5\;\mathrm{mm}}$ for calcifications in QRM_S, QRM_M and QRM_L (from top to bottom) with density of (a) $\frac{200\;\mathrm{mg}\;\mathrm{HA}}{cm^{3}}$, (b) $\frac{400\;\mathrm{mg}\;\mathrm{HA}}{cm^{3}}$, (c) $\frac{800\;\mathrm{mg}\;\mathrm{HA}}{cm^{3}}$.

Figure 8 demonstrates an axial plane of $I^{z}$, $\sigma_{\mathrm{CNN}}^{z}$, $I_{\mathrm{denoise}}^{z}$ (*z* = 0.5, 1.0, 1.5 mm) and $I^{3\;\mathrm{mm}}$ for QRM_S. Figure 9 presents the corresponding outcomes for QRM_M, while Figure 10 displays the results for QRM_L. The RMSE and PSNR between $I^{3\;\mathrm{mm}}$ and $I_{\mathrm{denoise}}^{z}$ for QRM phantoms are shown in Supplement 1. Figure 11 shows the Dice similarity coefficients calculated by comparing the calcifications pixels in $I^{3\;\mathrm{mm}}$ with those in $I^{z}$ and $I_{\mathrm{denoise}}^{z}$ (*z* = 0.5, 1.0, 1.5 mm) for calcifications in three anthropomorphic thorax phantoms with density of $\frac{200\;\mathrm{mg}\;\mathrm{HA}}{cm^{3}}$, $\frac{400\;\mathrm{mg}\;\mathrm{HA}}{cm^{3}}$, $\frac{800\;\mathrm{mg}\;\mathrm{HA}}{cm^{3}}$. Table 2 summaries the analysis results of HU values within background and calcification ROIs on $I^{3\;\mathrm{mm}}$, $I^{z}$ and $I_{\mathrm{denoise}}^{z}$ (*z* = 0.5, 1.0, 1.5 mm) for three electron density phantoms. The *p*‐values were acquired by conducting a two‐sample *t*‐test to assess the disparity in HU values between $I^{3\;\mathrm{mm}}$ and the remaining images.

**TABLE 2:** Analysis results of HU values within background and calcification ROIs for QRM_S, QRM_M, QRM_L.

		$\;I^{3\;\mathrm{mm}}$		$\;I^{0.5\;\mathrm{mm}}$			$\;I^{1.0\;\mathrm{mm}}$			$\;I^{1.5\;\mathrm{mm}}$			${\;I}_{\mathrm{denoise}}^{0.5\;\mathrm{mm}}$			${\;I}_{\mathrm{denoise}}^{1.0\;\mathrm{mm}}$			${\;I}_{\mathrm{denoise}}^{1.5\;\mathrm{mm}}$		
		Mean (HU)	SD (HU)	Mean (HU)	SD (HU)	*p*‐Value	Mean (HU)	SD (HU)	*p‐*Value	Mean (HU)	SD (HU)	*p*‐Value	Mean (HU)	SD (HU)	*p*‐Value	Mean (HU)	SD (HU)	*p*‐ Value	Mean (HU)	SD (HU)	*p*‐ Value
QRM_S	Background	41.65	22.77	41.70	48.73	0.90	41.72	37.89	0.83	41.74	30.89	0.75	41.61	19.94	0.83	41.05	19.12	0.01	41.20	19.72	0.04
	$\frac{200\;\mathrm{mg}\;\mathrm{HA}}{cm^{3}}$	243.41	52.49	248.56	63.12	0.46	256.17	67.28	0.08	256.56	62.94	0.06	219.73	49.86	^*^	230.21	55.76	0.04	238.67	55.99	0.46
	$\frac{400\;\mathrm{mg}\;\mathrm{HA}}{cm^{3}}$	372.45	168.43	382.48	163.82	0.51	377.56	165.63	0.74	373.24	166.89	0.96	375.87	152.54	0.82	367.49	155.05	0.74	363.73	159.81	0.56
	$\frac{800\;\mathrm{mg}\;\mathrm{HA}}{cm^{3}}$	611.27	383.27	618.06	385.29	0.83	622.14	387.07	0.73	606.71	382.79	0.89	617.07	357.76	0.85	628.07	375.05	0.59	606.67	372.55	0.88
QRM_M	Background	40.26	21.77	40.07	46.79	0.64	40.11	36.25	0.63	40.32	29.71	0.84	40.20	19.33	0.80	39.69	18.63	0.01	39.81	19.04	0.04
	$\frac{200\;\mathrm{mg}\;\mathrm{HA}}{cm^{3}}$	231.04	56.89	251.64	68.11	0.01	247.50	56.87	0.02	237.02	54.13	0.39	219.56	47.16	0.08	222.99	47.79	0.22	222.49	48.42	0.19
	$\frac{400\;\mathrm{mg}\;\mathrm{HA}}{cm^{3}}$	368.88	146.18	382.59	148.77	0.33	382.48	146.64	0.33	380.65	147.63	0.40	366.74	143.43	0.88	366.37	139.73	0.85	365.81	140.46	0.82
	$\frac{800\;\mathrm{mg}\;\mathrm{HA}}{cm^{3}}$	591.05	353.91	605.40	353.69	0.63	603.92	356.67	0.67	602.45	355.67	0.71	594.72	329.81	0.90	590.47	340.61	0.98	593.69	346.41	0.93
QRM_L	Background	47.33	21.33	47.30	46.47	0.92	47.57	34.47	0.40	47.26	29.27	0.80	47.42	18.85	0.65	47.06	17.62	0.17	46.86	18.80	0.02
	$\frac{200\;\mathrm{mg}\;\mathrm{HA}}{cm^{3}}$	225.72	42.59	238.26	60.70	0.06	234.70	47.89	0.12	234.17	45.74	0.13	213.66	53.49	0.05	213.23	44.72	0.03	225.27	42.37	0.93
	$\frac{400\;\mathrm{mg}\;\mathrm{HA}}{cm^{3}}$	348.57	144.18	367.84	141.24	0.15	367.14	145.46	0.18	363.57	142.65	0.27	354.33	137.49	0.67	351.53	141.17	0.83	353.13	139.03	0.73
	$\frac{800\;\mathrm{mg}\;\mathrm{HA}}{cm^{3}}$	566.63	320.64	581.63	326.44	0.59	580.33	328.22	0.62	581.83	326.25	0.58	563.23	307.02	0.90	570.16	319.13	0.90	568.83	318.48	0.94

Abbreviation: HU, Hounsfield units; ROI, region of interest; SD, standard deviation.

^*^ The asterisk indicates a *p*‐value < 0.01.

### 3.3 Evaluation with patient scans

**FIGURE 12:**
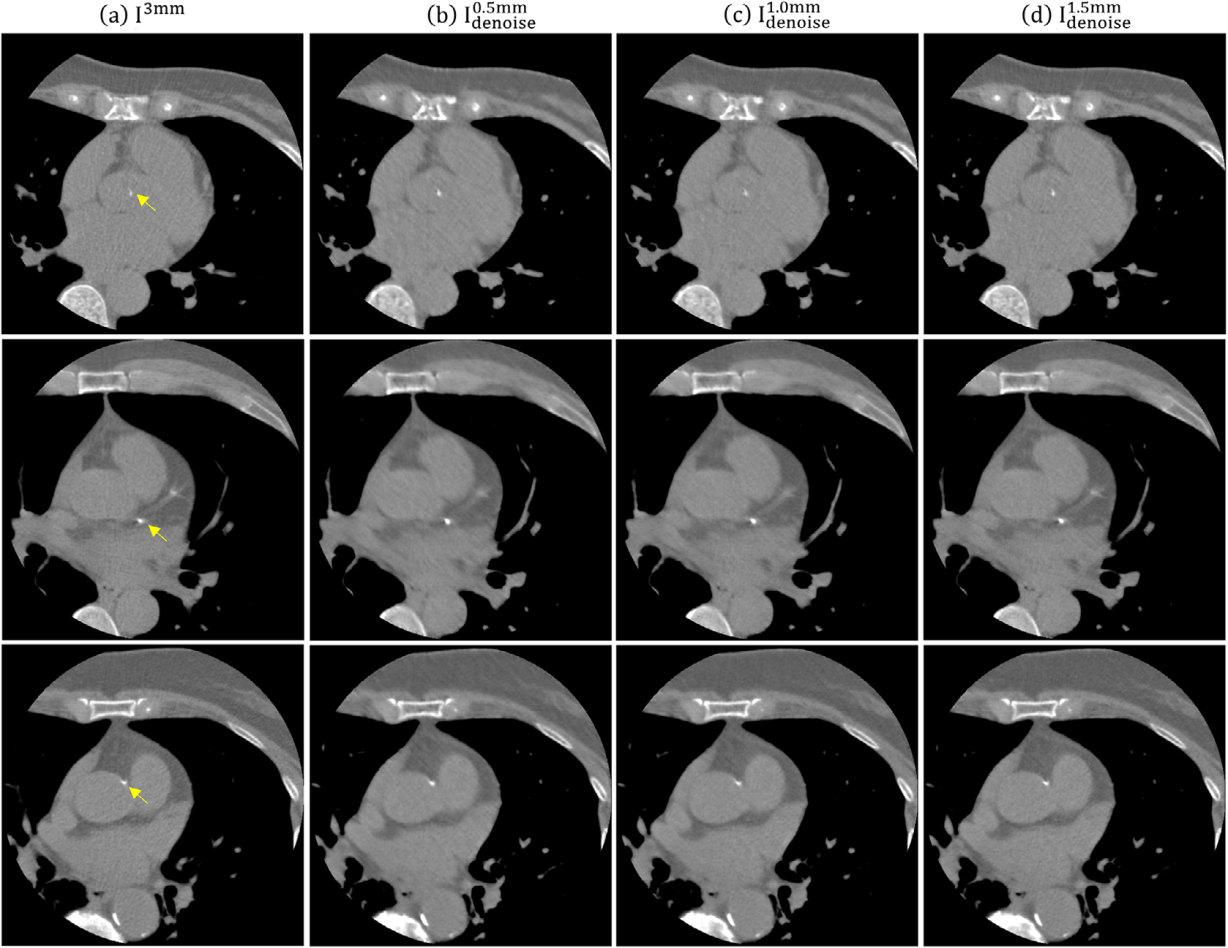
Axial plane (window level/window width = 0/1000 HU) of (a) $I^{3\;\mathrm{mm}}$, (b) $I_{\mathrm{denoise}}^{0.5\;\mathrm{mm}}$, (c) $I_{\mathrm{denoise}}^{1.0\;\mathrm{mm}}$, (d) $I_{\mathrm{denoise}}^{1.5\;\mathrm{mm}}$ for patient #1, patient #2 and patient #3 (from top to bottom). HU, Hounsfield units.

**FIGURE 13:**
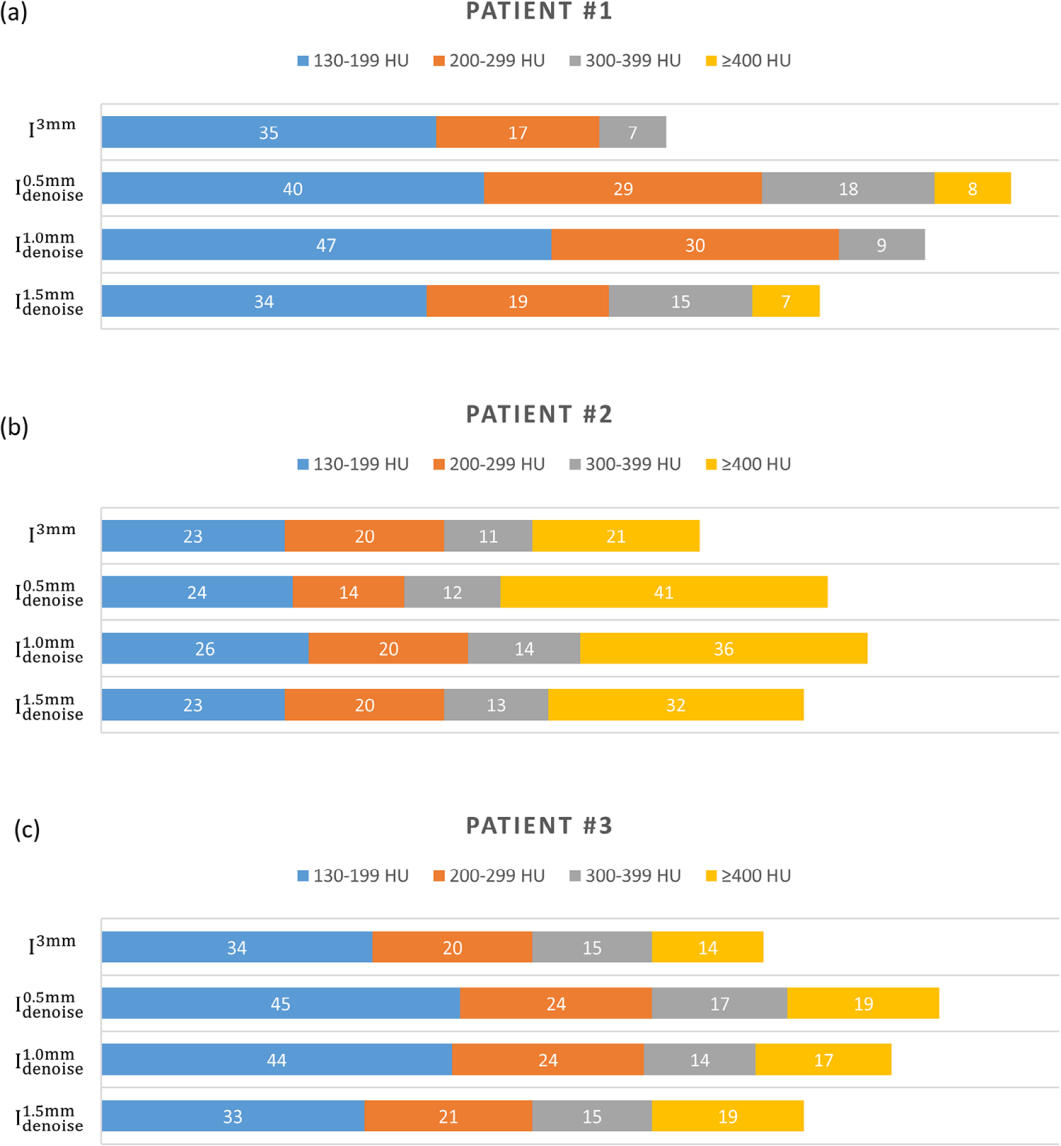
Comparing the number of calcification pixels for (a) patient #1, (b) patient #2, (c) patient #3.

Figure 12 demonstrates an axial plane of $I^{3\;\mathrm{mm}}$ and $I_{\mathrm{denoise}}^{z}$ (*z* = 0.5, 1.0, 1.5 mm) for three individual patients. The CTDI_vol_ was 2.6, 5.2, 7.6 mGy for patient #1 (body mass index (BMI) = 27 kg/m^2^), patient #2 (BMI = 31.5 kg/m^2^), patient #3 (BMI = 38.2 kg/m^2^), respectively, which were resulted from a CAC scan with tube current of 120, 210, 300 mA. The pixels of the calcifications indicted by yellow arrow were classified into 4 HU groups, and the results were shown in Figure 13. For the first patient, the total number of calcification pixels detected on $I^{3\;\mathrm{mm}}$, $I_{\mathrm{denoise}}^{0.5\;\mathrm{mm}}$, $I_{\mathrm{denoise}}^{1.0\;\mathrm{mm}}$, $I_{\mathrm{denoise}}^{1.5\;\mathrm{mm}}$ were 59, 95, 86, 65, respectively. For the second patient, the total number of calcification pixels detected on $I^{3\;\mathrm{mm}}$, $I_{\mathrm{denoise}}^{0.5\;\mathrm{mm}}$, $I_{\mathrm{denoise}}^{1.0\;\mathrm{mm}}$, $I_{\mathrm{denoise}}^{1.5\;\mathrm{mm}}$ were 75, 91, 96, 88, respectively. For the third patient, the total number of calcification pixels detected on $I^{3\;\mathrm{mm}}$, $I_{\mathrm{denoise}}^{0.5\;\mathrm{mm}}$, $I_{\mathrm{denoise}}^{1.0\;\mathrm{mm}}$, $I_{\mathrm{denoise}}^{1.5\;\mathrm{mm}}$ were 83, 105, 99, 88, respectively. An axial plane of $I^{z}$, $\sigma_{\mathrm{CNN}}^{z}$, $I_{\mathrm{denoise}}^{z}$ and $I^{3\;\mathrm{mm}}$ for three individual patients are shown in Supplement 2–4.

## 4 DISCUSSION

Several deep learning methods have been used to reduce the quantum noise in CT imaging, including discriminative models, generative models, and hybrid models.^16^, ^17^, ^18^, ^19^ The DenseNet model used in this work was based on the discriminative approach that represents bottom‐up execution to define a decision boundary that divides the feature space into areas containing feature vectors belong to the same class.^22^ The discriminative models have been demonstrated to be able to reduce the streaking artifacts tremendously while preserving the fine structures in the processed CT images.^17^, ^18^, ^19^ However, it has also been reported that the discriminative models would cause over‐smooth changes in the image due to the least squares objective function.^16^, ^17^ Based on our experience, when the electron density phantoms were used to acquire datasets for model training, the CNN output images were quite blurry if DenseNet was trained with $I^{z}$ (*z* = 0.5, 1.0, 1.5 mm) as input and $I^{3\;\mathrm{mm}}$ as label. In our opinion, this phenomenon may be due to the lack of high‐resolution insert in the electron density phantoms. Usually, model training was conducted by using datasets acquired from similar scenarios that is encountered in the denoising task. Hence, the degradation in spatial resolution was not observed in the denoising method trained with images acquired with the anthropomorphic thorax phantoms. Divel et al. have proposed an image domain noise insertion method for CT images, in which white noise was filtered, scaled and added to the image to simulate low‐dose scans.^23^ Their work inspired us to start from the following aspects: (1) it may be possible to conduct image denoising by subtracting quantum noise from images; (2) quantum noise contains less anatomical details compared to CT images. Hence, the workflow shown in Figure 2 was proposed to reduce quantum noise in thin‐sliced CAC scans, where the input and label of DenseNet were $\sigma_{\mathrm{pseudo}}^{z}$ and $\sigma_{\mathrm{real}}^{z}$ (*z* = 0.5, 1.0, 1.5 mm), respectively.

For the validation with electron density phantoms, $\sigma_{\mathrm{CNN}}^{0.5\;\mathrm{mm}}$ shows the highest statistical fluctuations, followed by $\sigma_{\mathrm{CNN}}^{1.0\;\mathrm{mm}}$ and $\sigma_{\mathrm{CNN}}^{1.5\;\mathrm{mm}}$. Based on naked eye observation, there was a noticeable difference in overall texture between $I^{3\;\mathrm{mm}}$ and $I_{\mathrm{denoise}}^{0.5\;\mathrm{mm}}$, but the difference was less obvious between $I^{3\;\mathrm{mm}}$ and $I_{\mathrm{denoise}}^{1.0\;\mathrm{mm}}$ and was barely discernable between $I^{3\;\mathrm{mm}}$ and $I_{\mathrm{denoise}}^{1.5\;\mathrm{mm}}$. With regard to the results of ROI analysis, the SD values measured from $I_{\mathrm{denoise}}^{z}$ were lower than those from I^z^ (*z* = 0.5, 1.0, 1.5 mm), while the largest difference in mean value between I^z^ and $I_{\mathrm{denoise}}^{z}$ was 3.09 HU. No statistically significant difference was found in CIRS_S and CIRS_L. As for CIRS_M, statistically significant difference was found when comparing the HU values of bone insert with HA density of 200 mg/cc between $I^{3\;\mathrm{mm}}$ and $I_{\mathrm{denoise}}^{0.5\;\mathrm{mm}}$.

For the validation with anthropomorphic thorax phantoms, $\sigma_{\mathrm{CNN}}^{0.5\;\mathrm{mm}}$ shows the highest statistical fluctuations, followed by $\sigma_{\mathrm{CNN}}^{1.0\;\mathrm{mm}}$ and $\sigma_{\mathrm{CNN}}^{1.5\;\mathrm{mm}}$. Based on naked eye observation, the difference in background texture was noticeable between $I^{3\;\mathrm{mm}}$ and $I_{\mathrm{denoise}}^{0.5\;\mathrm{mm}}$, which was less obvious between $I^{3\;\mathrm{mm}}$ and $I_{\mathrm{denoise}}^{1.0\;\mathrm{mm}}$ and was barely discernable between $I^{3\;\mathrm{mm}}$ and $I_{\mathrm{denoise}}^{1.5\;\mathrm{mm}}$. The calcifications with density of $\frac{400\;\mathrm{mg}\;\mathrm{HA}}{cm^{3}}$ and $\frac{800\;\mathrm{mg}\;\mathrm{HA}}{cm^{3}}$ identified on $I_{\mathrm{denoise}}^{z}$ (*z* = 0.5, 1.0 and 1.5 mm) have little differences in terms of shape and sharpness when compared to those identified on $I^{3\;\mathrm{mm}}$. As for the calcifications with density of $\frac{200\;\mathrm{mg}\;\mathrm{HA}}{cm^{3}}$, calcification blur was observed in $I_{\mathrm{denoise}}^{0.5\;\mathrm{mm}}$, which was less obvious in $I_{\mathrm{denoise}}^{1.0\;\mathrm{mm}}$ and was barely discernable in $I_{\mathrm{denoise}}^{1.5\;\mathrm{mm}}$. The Dice similarity coefficients for calcifications with density of $\frac{200\;\mathrm{mg}\;\mathrm{HA}}{cm^{3}}$ obtained by comparing $I^{3\;\mathrm{mm}}$ and $I_{\mathrm{denoise}}^{1.5\;\mathrm{mm}}$ were usually higher than those obtained by comparing $I^{3\;\mathrm{mm}}$ and $I_{\mathrm{denoise}}^{1.0\;\mathrm{mm}}$ or comparing $I^{3\;\mathrm{mm}}$ and $I_{\mathrm{denoise}}^{0.5\;\mathrm{mm}}$. This phenomenon was also found in the calcifications with density of $\frac{400\;\mathrm{mg}\;\mathrm{HA}}{cm^{3}}$ and $\frac{800\;\mathrm{mg}\;\mathrm{HA}}{cm^{3}}$. With regard to the results of ROI analysis, the SD values measured from $I_{\mathrm{denoise}}^{z}$ were lower than those from I^z^ (*z* = 0.5, 1.0, 1.5 mm). No statistically significant difference was found in any QRM phantom at the background region. As for the calcifications, there was no statistically significant difference in QRM_M and QRM_L, while statistically significant difference was observed in QRM_S when comparing the HU values of calcifications with density of $\frac{200\;\mathrm{mg}\;\mathrm{HA}}{cm^{3}}$ identified on $I^{3\;\mathrm{mm}}$ to those on $I_{\mathrm{denoise}}^{0.5\;\mathrm{mm}}$.

By means of phantom studies, the proposed method was validated effective in reducing quantum noise in I^1.5mm^ without causing significant texture change or variation in HU values. In order to further confirm this finding, the proposed method underwent evaluation using actual patient data. Based on naked eye observation, over‐smooth changes were observed at the background region in $I_{\mathrm{denoise}}^{0.5\;\mathrm{mm}}$. This phenomenon was less obvious in $I_{\mathrm{denoise}}^{1.0\;\mathrm{mm}}$, while no serious texture changes were observed in $I_{\mathrm{denoise}}^{1.5\;\mathrm{mm}}$. The calcifications were more clear on $I_{\mathrm{denoise}}^{z}$ (*z* = 0.5, 1.0, 1.5 mm) than on $I^{3\;\mathrm{mm}}$, which can be confirm by the number of detected calcification pixels. Overall, this work demonstrated that the electron density phantoms can be used to generate training data for the proposed CNN‐based denoising method to reduce quantum noise for CAC scans reconstructed with 1.5‐mm slice thickness. Because anthropomorphic phantom is not a necessity, our method could make image denoising more practical in routine clinical practice. However, more clinical trials are necessary to provide evidence for the effectiveness of the proposed method.

## 5 CONCLUSION

This work proposed a CNN‐based method trained with images acquired with electron density phantoms to reduce quantum noise for CAC scans reconstructed with slice thickness less than 3 mm. By means of phantom studies, the proposed method was proven effective in reducing quantum noise in I^1.5mm^ without causing significant texture change or variation in HU values. With regard to patient study, the calcifications were more clear on $I_{\mathrm{denoise}}^{z}$ (*z* = 0.5, 1.0, 1.5 mm) than on $I^{3\;\mathrm{mm}}$, while over‐smooth changes were not observed in $I_{\mathrm{denoise}}^{1.5\;\mathrm{mm}}$. Our results demonstrated that the electron density phantoms can be used to generate training data for the proposed CNN‐based denoising method to reduce quantum noise for CAC scans reconstructed with 1.5‐mm slice thickness. Because anthropomorphic phantom is not a necessity, our method could make image denoising more practical in routine clinical practice.

## AUTHOR CONTRIBUTIONS

**Ching‐Ching Yang**: Conceptualization; methodology; software; validation; formal analysis; investigation; resources; data curation; writing; visualization; supervision. **Kuei‐Yuan Hou**: Formal analysis; resources; data curation; writing; project administration.

## CONFLICT OF INTEREST STATEMENT

The authors declare no conflicts of interest.

## Supporting information

Supporting Information
