# Supplementary material for: A CNN‐based denoising method trained with images acquired with electron density phantoms for thin‐sliced coronary artery calcium scans
Source: J Appl Clin Med Phys. 2024 Feb 12;25(3):e14287. doi: 10.1002/acm2.14287 (PMC10930013; doi:10.1002/acm2.14287)
Supplement: Supplementary file 1 — Supporting Information [file ACM2-25-e14287-s001.docx]

Supplement legends

Supplement 1. RMSE (solid line, left axis) and PSNR (dashed line, right axis) between (a) $I^{3mm}$and $I_{\mathrm{denoise}}^{0.5mm}$, (b) $I^{3mm}$and $I_{\mathrm{denoise}}^{1.0mm}$, (c) $I^{3mm}$and $I_{\mathrm{denoise}}^{1.5mm}$ under different training iterations for QRM_S, QRM_M, QRM_L (from top to bottom)


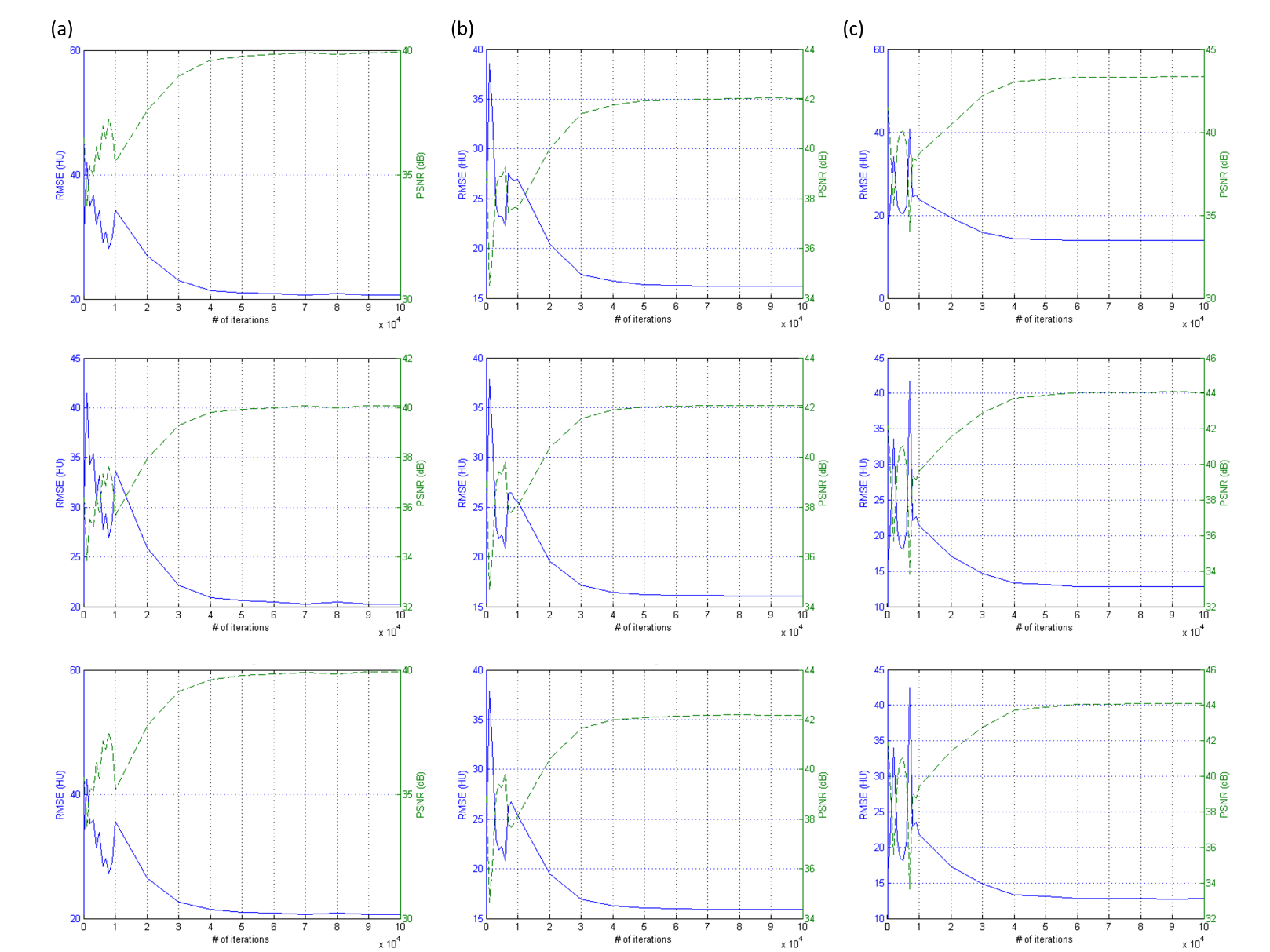


Supplement 2. Axial plane of (a) $I^{0.5mm}$, (b) $I^{1.0\mathrm{mm}}$, (c) $I^{1.5mm}$, (d) $I^{3mm}$, (e) $\sigma_{\mathrm{CNN}}^{0.5mm}$, (f) $\sigma_{\mathrm{CNN}}^{1.0mm}$, (g) $\sigma_{\mathrm{CNN}}^{1.5mm}$, (h) $I_{\mathrm{denoise}}^{0.5mm}$, (i) $I_{\mathrm{denoise}}^{1.0mm}$, (j) $I_{\mathrm{denoise}}^{1.5mm}$ for patient #1 (window level/window width = 0/1000 HU for (a)-(d), (h)-(j) and 0/340 HU for (e)-(g)).


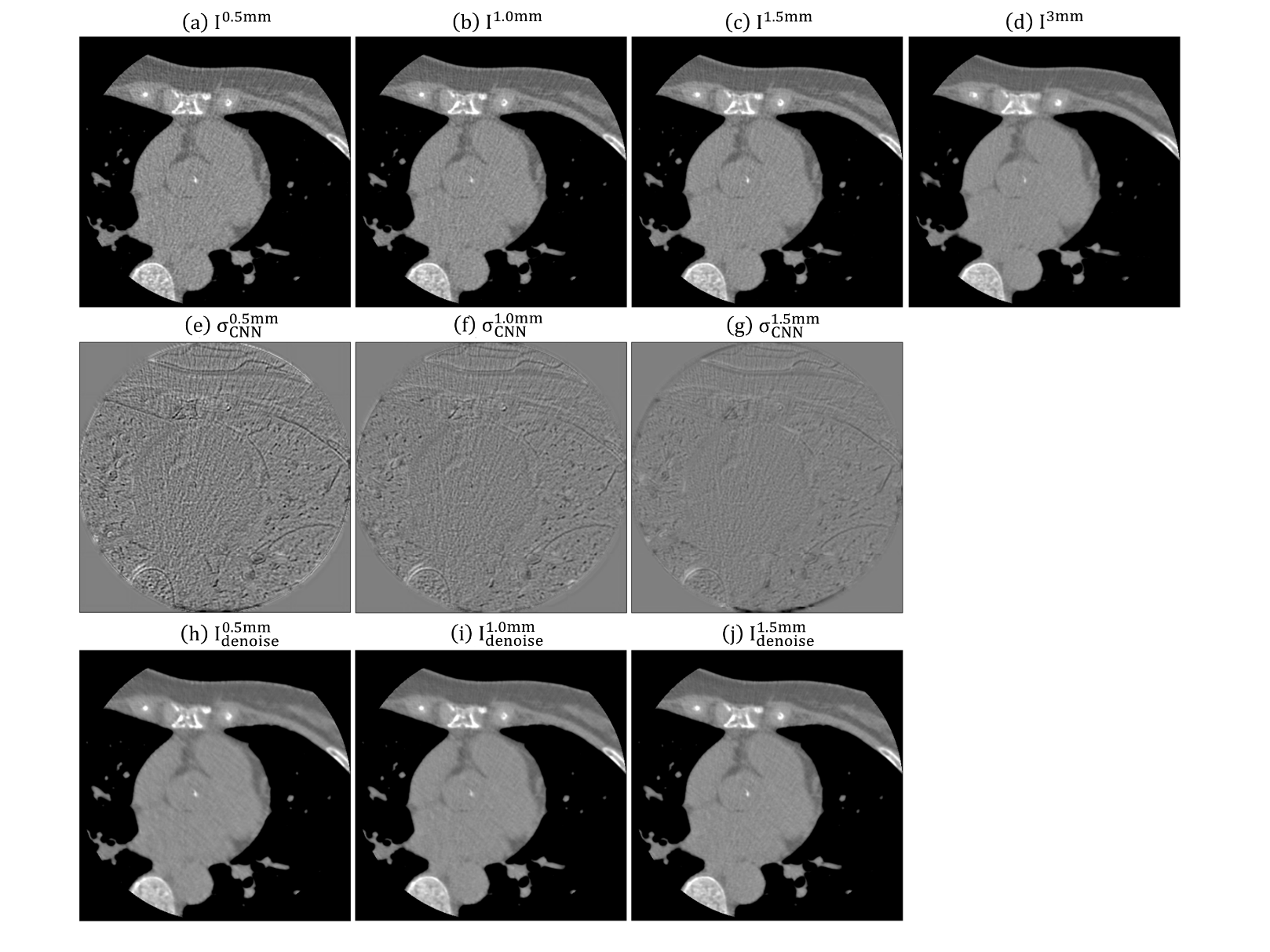


Supplement 3. Axial plane of (a) $I^{0.5mm}$, (b) $I^{1.0\mathrm{mm}}$, (c) $I^{1.5mm}$, (d) $I^{3mm}$, (e) $\sigma_{\mathrm{CNN}}^{0.5mm}$, (f) $\sigma_{\mathrm{CNN}}^{1.0mm}$, (g) $\sigma_{\mathrm{CNN}}^{1.5mm}$, (h) $I_{\mathrm{denoise}}^{0.5mm}$, (i) $I_{\mathrm{denoise}}^{1.0mm}$, (j) $I_{\mathrm{denoise}}^{1.5mm}$ for patient #2 (window level/window width = 0/1000 HU for (a)-(d), (h)-(j) and 0/340 HU for (e)-(g)).


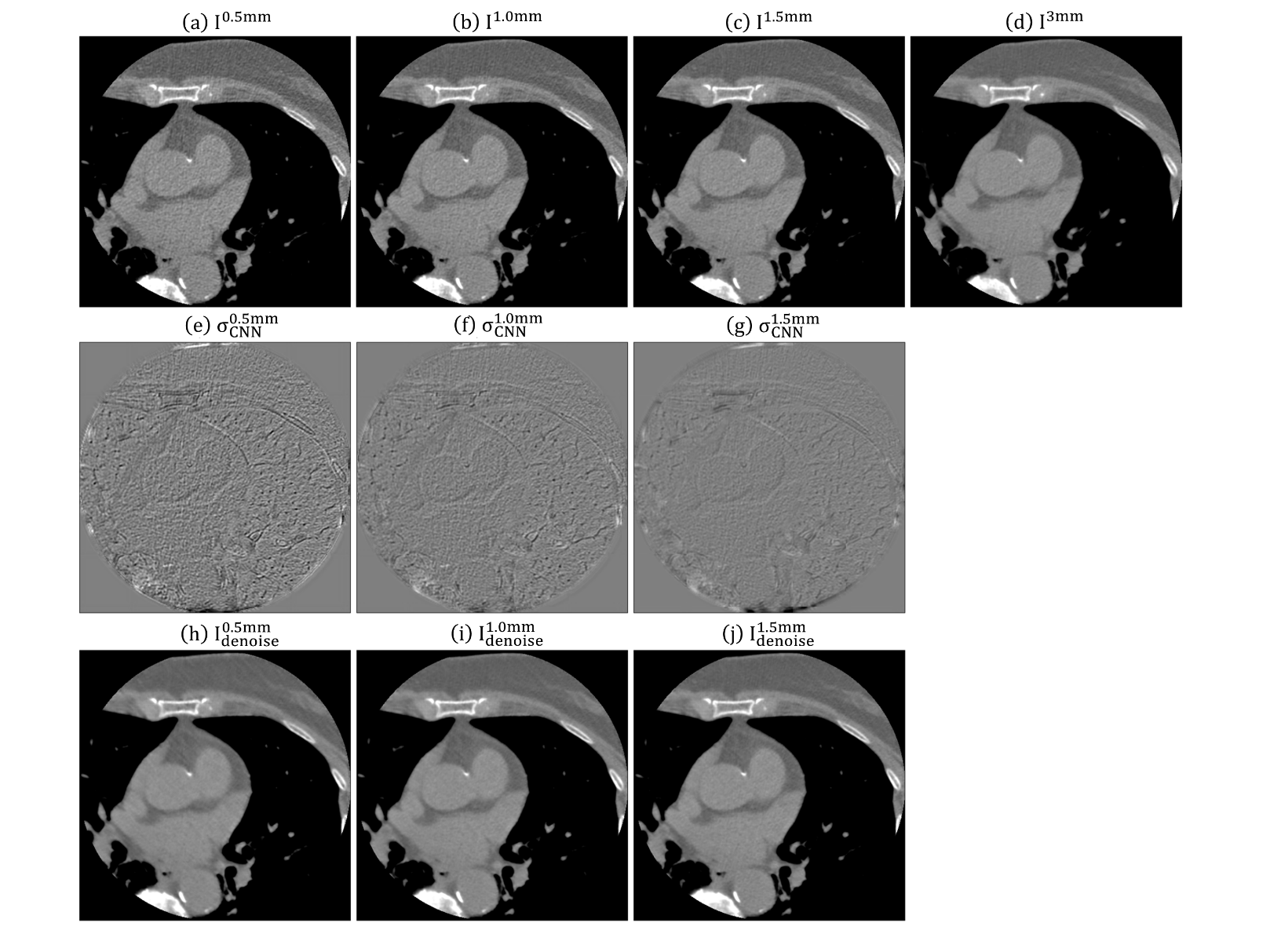


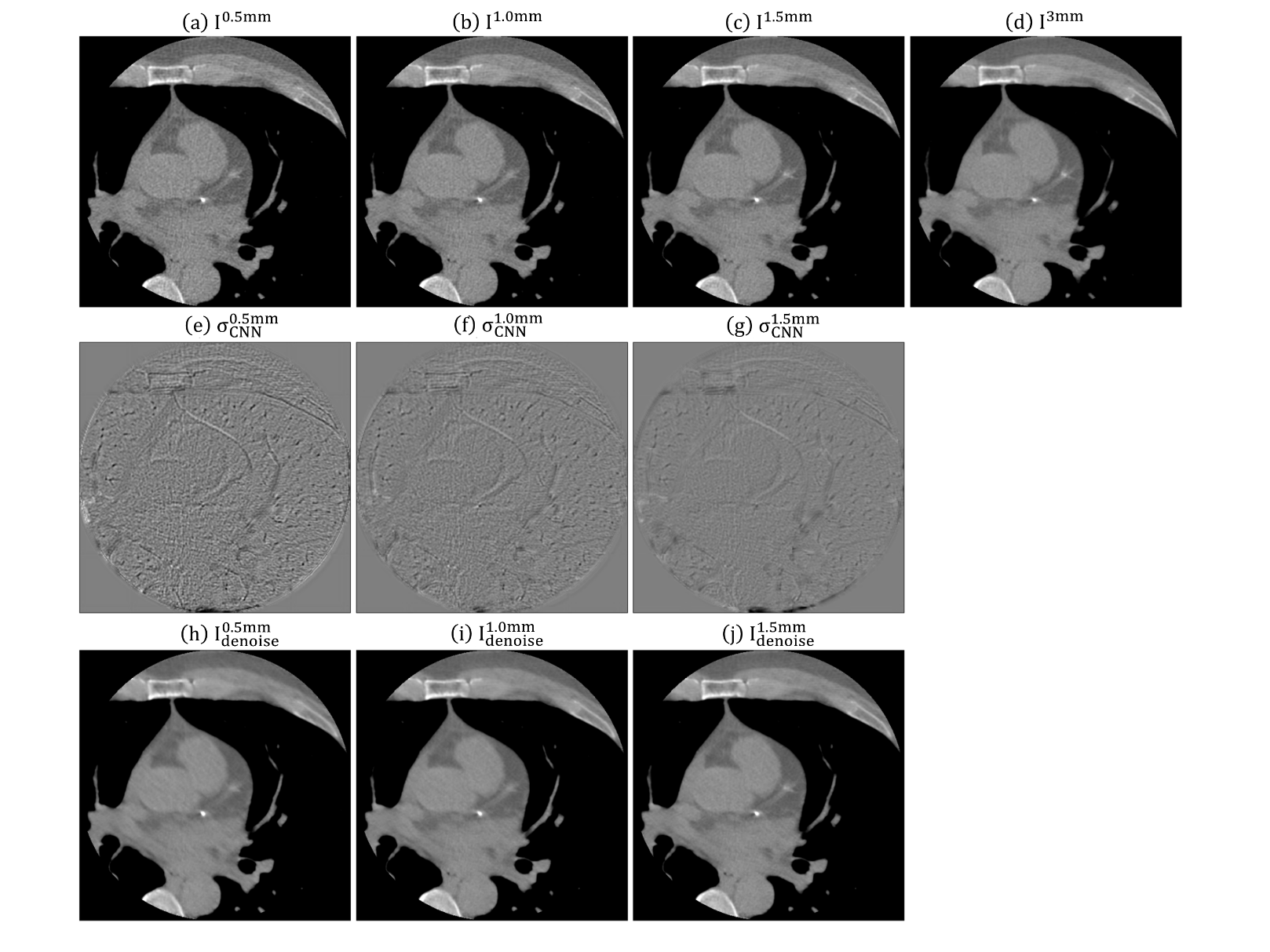


Supplement 4. Axial plane of (a) $I^{0.5mm}$, (b) $I^{1.0\mathrm{mm}}$, (c) $I^{1.5mm}$, (d) $I^{3mm}$, (e) $\sigma_{\mathrm{CNN}}^{0.5mm}$, (f) $\sigma_{\mathrm{CNN}}^{1.0mm}$, (g) $\sigma_{\mathrm{CNN}}^{1.5mm}$, (h) $I_{\mathrm{denoise}}^{0.5mm}$, (i) $I_{\mathrm{denoise}}^{1.0mm}$, (j) $I_{\mathrm{denoise}}^{1.5mm}$ for patient #3 (window level/window width = 0/1000 HU for (a)-(d), (h)-(j) and 0/340 HU for (e)-(g)).
